# Transcriptome Analysis of Phycocyanin-Mediated Inhibitory Functions on Non-Small Cell Lung Cancer A549 Cell Growth

**DOI:** 10.3390/md16120511

**Published:** 2018-12-15

**Authors:** Shuai Hao, Shuang Li, Jing Wang, Lei Zhao, Yan Yan, Qi Cao, Tingting Wu, Liyun Liu, Chengtao Wang

**Affiliations:** 1 Beijing Advanced Innovation Center for Food Nutrition and Human Health, Beijing Engineering and Technology Research Center of Food Additives, Beijing Technology and Business University, Beijing 100048, China; haoshuai@btbu.edu.cn (S.H.); lishuangldw@163.com (S.L.); trotwj960@163.com (J.W.); zhaolei@th.btbu.edu.cn (L.Z.); shmilyhs321@163.com (Y.Y.); CJ118214@163.com (Q.C.); m18810529269@163.com (T.W.); 2 State Key Laboratory of Infection Disease Prevention and Control, National Institute for Communicable Disease Control and Prevention, Collaborative Innovation Center for Diagnosis and Treatment of Infectious Disease, Chinese Center for Disease Control and Prevention, Beijing 102206, China

**Keywords:** phycocyanin, non-small cell lung cancer, A549 cells, cell growth, RNA-seq, qRT-PCR

## Abstract

Phycocyanin (PC), derived from cyanobacteria and *Spirulina* cells, is a type of natural antineoplastic marine protein. It has been reported that phycocyanin exerts an antitumor function in non-small cell lung cancer (NSCLC) cells, but the underlying mechanism has not been elucidated. In this research, a transcriptome study was performed to investigate the regulatory mechanisms of phycocyanin on human NSCLC A549 cells. The survival rate and proliferation ability of A549 cells were markedly reduced by phycocyanin, along with abnormal morphologic changes. The transcriptome analysis showed that 2970 genes were differentially expressed after phycocyanin treatment in A549 cells, including 1431 down-regulated and 1539 up-regulated genes. Gene ontology and KEGG analysis suggested that some classical pathways, such as Wnt, NF-κB, and PI3K-AKT signaling, were significantly enriched. Strikingly, protein–protein interaction (PPI) analysis showed that ubiquitin-C (UBC) occupied the highest degree (the highest number of interactions) in differential genes, indicating that it might play a key role in the phycocyanin-mediated regulatory process in A549 cells. Moreover, qRT-PCR results showed consistent expression trends of differential genes with transcriptome analysis. Consequently, this study has provided a theoretical basis for regulation of phycocyanin in A549 cells, which lays a foundation for the treatment of NSCLC.

## 1. Introduction

Lung cancer is the leading cause of cancer-related death in males, and the second leading cause of death in females worldwide [1]. Non-small cell lung cancer (NSCLC) accounts for over 80% of all lung cancer cases, with the characteristics of high mortality, low cure rate and strong metastasis. Nowadays, surgery is the mainstream therapy method for NSCLC, while other treatments, such as chemotherapy combined with paclitaxel and immune-based therapies, are also reported to improve survival rates [2,3]. However, the postoperative recurrence rate of NSCLC is still very high despite most patients being sensitive to the first-line therapy process [4]. Recently, investigators have been focusing on exploring the underlying mechanisms of new natural products with antineoplastic activities, which will provide valuable information on NSCLC treatment.

Phycocyanin (PC), a marine natural blue photosynthetic pigment protein purified from cyanobacteria and *Spirulina* cells, is one of the accepted natural functional food additives around the world [5]. Studies have shown that phycocyanin exerts multiple biological functions including antineoplastic, antioxidant, immunomodulation, and anti-inflammatory activities [6,7,8,9], and it is thus attracting more attention regarding its development and utilization [10]. It has been reported that phycocyanin exerts anti-tumor activities in multiple cancer cells including breast cancer [6], pancreatic cancer [11], colon cancer [12], liver cancer [13], and gastric cancer [14]. Phycocyanin has great potential as NSCLC treatment. Li et al. have reported that phycocyanin could remarkably inhibit the growth of NSCLC A549 cells in vivo and in vitro, which also has a synergistic antitumor effect with all-trans retinoic acid [15,16]. Baudelet et al. discovered that glaucophyte *Cyanophora paradoxa* extracts could significantly inhibit the growth of three cancer cell lines, including A549 cells [17]. Bingula et al. reported that phycocyanin and betaine have a synergistic inhibiting effect on the viability of A549 cells [18]. In addition, besides A549 cells, our previous work indicated that phycocyanin could inhibit the proliferation and induce the apoptosis in three other types of NSCLC cells (H1299, H460, and LTEP-a2) [19]. It is worth mentioning that while most studies reported the function of phycocyanin on NSCLC cells at the phenotypic level, the underlying mechanism still remains unclear. Therefore, further exploration of the mechanism of phycocyanin on NSCLC cells is critically needed.

High-throughput sequencing has revolutionized genomics research with its low cost and ultra-high data output. RNA sequencing (RNA-seq) is a high-throughput method that can be used to determine transcript abundance [20] and identify novel transcriptionally active regions [21]. Nowadays, RNA-seq analysis has become an effective way to study functional genes and mechanisms in anti-tumor research. Jian et al. performed RNA-seq to investigate the molecular events involved in an interferon inducible p204-mediated anti-tumor process [22]. Their results showed that numerous cytokines, receptors, and adhesion molecules were significantly induced after p204 knockout, which provided novel insights to the p204 network in anti-tumor response. Aldaz et al. compared the transcription differences between the wild type and breast cancer mice with p53 mutations [23] and discovered a series of new genes regulated by p53. It should be noted that there have been few reports on the mechanism of phycocyanin in tumor models via RNA-seq technique, except for Ying et al., who studied the inhibitory effect of phycocyanin on ovarian cancer SKOV-3 cells through transcriptome analysis. Their results suggested that vascular endothelial growth factor (VEGF), p53 signaling pathway might play an important biological role in phycocyanin treated cells [24]. In the present study, we investigated the potential mechanism of the phycocyanin-mediated NSCLC A549 cells inhibition process using transcriptome analysis for the first time. This study was expected to lay a theoretical foundation for the future treatment of NSCLC and the utilization of phycocyanin.

## 2. Results

### 2.1. Effect of Phycocyanin on the Morphology and Growth of the NSCLC A549 Cell Line

**Figure 1:**
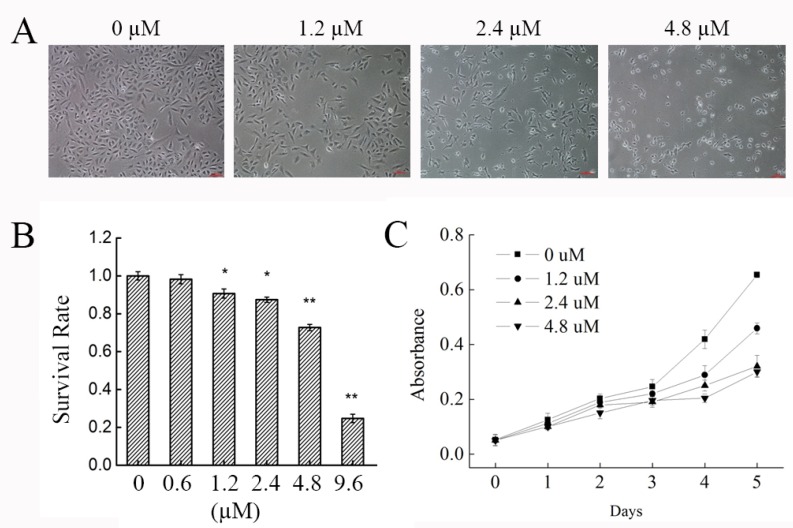
Effect of phycocyanin on the morphology and growth of the non-small cell lung cancer (NSCLC) A549 cell line. (**A**) Morphologic changes of A549 cells induced by different concentrations (0, 1.2, 2.4, and 4.8 µM) of phycocyanin (100×) treatment for 48 h. Scale bars represent 100 µm. (**B**) Survival rate analysis of A549 cells after different concentrations (0, 0.6, 1.2, 2.4, 4.8, and 9.6 µM) of phycocyanin treatment for 48 h. (**C**) MTT analysis of A549 cell proliferation after 0, 1.2, 2.4, and 4.8 µM phycocyanin treatment. Four replicates were carried out in survival rate and proliferation assays (*n* = 4). Bars represent mean ± SD. *, *p* < 0.05; **, *p* < 0.01.

Phycocyanin is proven to be an effective cancer treatment as it can inhibit the growth of different tumor cells [15,25]. In the present study, to address the effects of phycocyanin on NSCLC cells, we confirmed the phenotypic experiments on A549 cells. Cells treated with phycocyanin showed noticeable morphologic changes including abnormal shape, weakened refractivity, shrinkage, reduced cell–cell junction and decreased reproducibility with an increase of phycocyanin dosage (Figure 1A). The inhibitory effects of phycocyanin on the viability and proliferation of A549 cells were determined. As shown in Figure 1B, compared with control cells, incubation with phycocyanin (0, 0.6, 1.2, 2.4, 4.8, and 9.6 µM) dose-dependently inhibited the viability of A549 cells. The IC50 of phycocyanin was 6.18 µM in A549 cells. The MTT assay showed that phycocyanin could significantly suppress the proliferation of A549 cells in a dose-dependent manner (Figure 1C), which is consistant with results in Li et al. and Bingula et al. [16,18].

### 2.2. RNA Sequencing Data Analysis: Quality Control, Assembly, and Mapping

Based on the phenotypic experiments of A549 cells, a 4.8 µM concentration of phycocyanin was chosen for further study. High-throughput sequencing was used for the systematic analysis of gene expressions in A549 cells transcriptome with or without phycocyanin exposure. Approximately 65.5 and 57.3 million raw reads were obtained from control and test groups, respectively (Table 1). After a check for read quality and removal of contamination, a huge proportion (94.5%) of high-quality clean reads (61 and 55.1 million for control and test groups, respectively) remained for assembly and further downstream analysis. The clean reads were mapped to the human reference genome and the detailed mapping output is summarized in Table 1. In all, the mapping ratios for the control and the treatment group were 94.40% and 94.05%, respectively, indicating high levels of gene expression in both groups.

### 2.3. Functional Annotation and Gene Ontology Classification

Functional annotation gives information on protein function annotation, Clusters of Orthologous Groups of proteins (COG) annotation and Gene Ontology (GO) annotation. The transcripts and genes were aligned with public protein databases such as KEGG, COG and GO. In total, there were 122,847 (61.66%) transcripts and 19,937 (10.01%) genes successfully annotated (Table 2). Most of the transcripts and genes were annotated using the KEGG database (52.52% and 6.66%, respectively) followed by COG (8.49% and 29%, respectively) and GO (35.2% and 9.64%, respectively).

The COG database contains orthologous proteins that were classified under several categories. The genes were aligned to the COG database to predict and classify their possible function. Supplementary Figure S1 shows that the genes and transcripts were assigned into 24 orthologous clusters in COG. Some genes may be assigned into several clusters in COG categories, while some were assigned to the same cluster but with different protein orthologous similarity. The majority of the genes and transcripts were distributed in general function prediction (1399 and 1847, respectively), followed by posttranslational modification, protein turnover, chaperones (607 and 831, respectively), and signal transduction mechanisms (384 and 547, respectively).

The genes were further annotated and classified under Gene Ontology. GO is an international standardized gene functional classification system. It has three ontologies including molecular function, cellular component and biological process. Supplementary Figure S2 shows the distribution of genes assigned in Gene Ontology. In total, there were 250,299 genes mapped to GO, with 29,208 genes assigned to molecular function, 93,803 genes assigned to cellular component, and 127,188 genes assigned to biological process. One gene may be assigned into several different GO terms.

Furthermore, the genes were annotated and classified by KEGG database, which has six signaling pathways, including Metabolism, Genetic Information Processing, Environmental Information Processing, Cellular Processes, Organismal System, and Human Diseases. Supplementary Figure S3 shows the distribution of genes assigned in KEGG. Genes assigned to signal transduction (1712) occupied the maximum proportion, followed by that assigned to cancers (1143) and immune system (980). It is known that phycocyanin has significant inhibition properties in different kinds of cancer [6,18,19], and the present research might provide useful information for phycocyanin-mediated lung cancer regulation studies.

### 2.4. Identification of Differentially Expressed Genes (DEGs)

**Figure 2:**
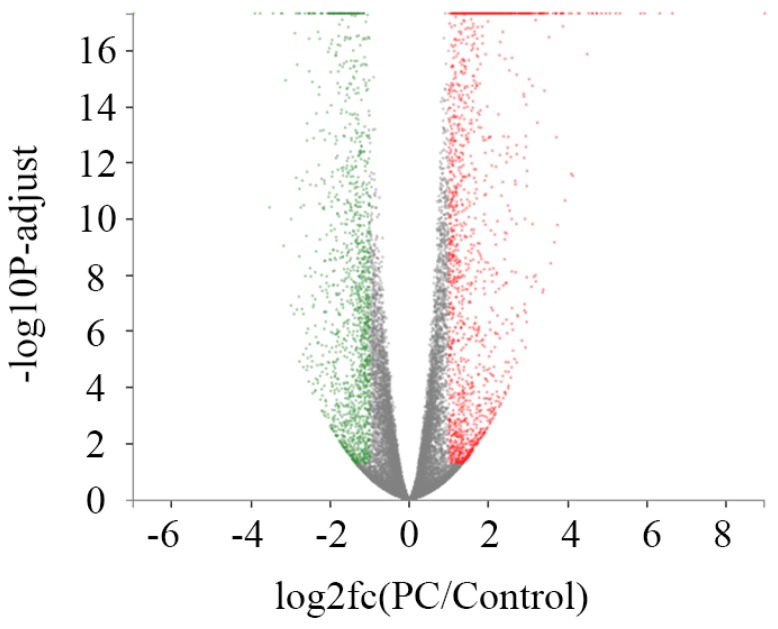
Differentially expressed genes (DEGs) between different treatment groups. The horizontal axis indicates expression changes (log) of the genes in different treatment groups while the vertical axis shows the differences of gene expression. The discrepancy was more significant with smaller *p* values and bigger −log10 (adjusted *p* value). Splashes were for different genes, among which grey dots were genes with no significant discrepancy, red dots were genes significantly up-regulated and green dots were significantly down-regulated genes. PC (phycocyanin): A549 cells with phycocyanin treatment for 48 h; Control: A549 cells without phycocyanin treatment.

A rigorous comparison at adjusted *p* ≤ 0.05 and log2FC fold change ≥ 1 (for up-regulation) or ≤ −1 (for down-regulation), was made to identify the number of DEGs for different groups (Figure 2). The list of DEGs, along with their fold changes and annotations, are presented in Supplementary Table S1. In total, gene expression analysis showed that 2970 genes were significantly differentially expressed, including 1539 up-regulated and 1431 down-regulated genes (Table 3). Functionalities of those genes included intracellular substance and energy metabolism, mitochondrial function, cell cycle, differentiation and apoptosis, protein oxidative phosphorylation and cell signal, RNA function, DNA repair and protein synthesis, and some other enzymes for metabolism.

### 2.5. Gene Ontology Analysis of Differentially Expressed Genes

**Figure 3:**
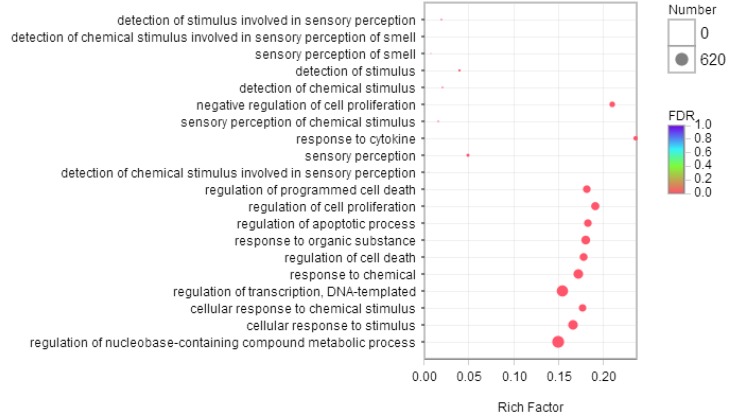
Bubble diagram of top 20 ranked GO terms of differentially expressed genes (DEGs). The vertical axis indicates GO terms and the horizontal axis represents the Rich factor. The enrichment degree was stronger with a bigger Rich factor. The size of dots indicates the number of genes in the GO term.

To characterize the functional consequences of gene expression changes caused by phycocyanin exposure, we performed GO enrichment analysis of 2970 DEGs based on the GO database. Figure 3 shows the top 20 ranked GO terms of DEGs. Response to cytokine occupied the strongest enrichment degree as it possessed the highest Rich factor (0.25), followed by negative regulation of cell proliferation (Rich factor 0.21). Interestingly, it was reported that phycocyanin had a growth inhibitory effect on multiple tumor cells [6,17,18,19], which was consistent with our results. In addition, regulation of nucleobase-containing compound metabolic process, regulation of transcription, DNA-templated, and cellular response to stimulus were the most abundant functional groups in most of the comparisons. GO enrichment analysis of DEGs showed that cellular metabolic process, cell communication, cell proliferation and cell programmed death were the main types in biological process, indicating that the cell process of A549 cells was the most influenced function by phycocyanin.

### 2.6. KEGG Pathway Analysis of Differentially Expressed Genes

**Figure 4:**
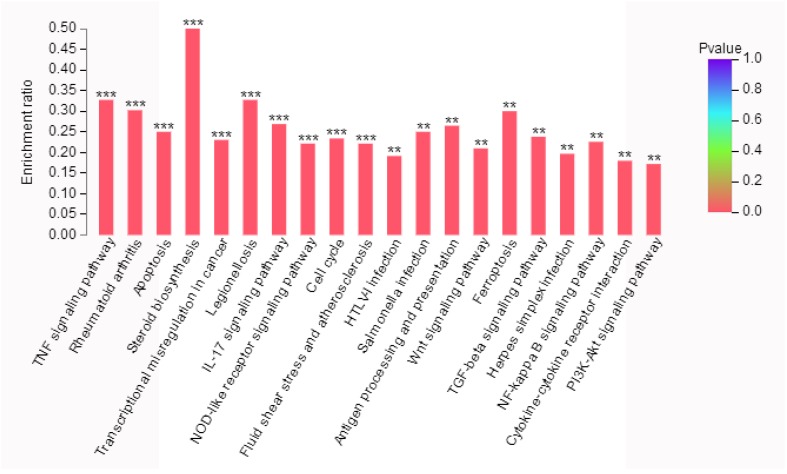
KEGG pathway enrichment analysis of differentially expressed genes (DEGs). The vertical axis indicates the enrichment ratio. The enrichment degree was stronger with a higher enrichment ratio. The horizontal axis indicates different pathways. Asterisks represent significant difference levels: **, *p* < 0.01; ***, *p* < 0.001.

In an organism, genes coordinate with each other to function biologically. Pathway analysis can help better understanding the biological function of genes. Through pathway significance enrichment analysis, biochemical metabolic and signal transduction pathways, in which differential genes participated, were determined. As a result, 50 metabolic pathways with significantly differential expressions (*p* < 0.05) were identified between treatment and control groups, of which 31 pathways showed the most significant difference (*p* < 0.01). Figure 4 shows the top 20 ranked significant pathways in KEGG. Many signal transduction pathways were significantly enriched, including the TNF signaling pathway, IL-17 signaling pathway, NOD-like receptor pathway, Wnt signaling pathway, TGF-beta signaling pathway, NF-κB signaling pathway, and PI3K-Akt pathway. In addition, biological process regulation like apoptosis, cell cycle and ferroptosis were significantly enriched in our result. These results could provide essential information on the investigation of phycocyanin in non-small cell lung cancer A549 cells.

### 2.7. Protein–Protein Interaction (PPI) Network Analysis of Differentially Expressed Genes

**Figure 5:**
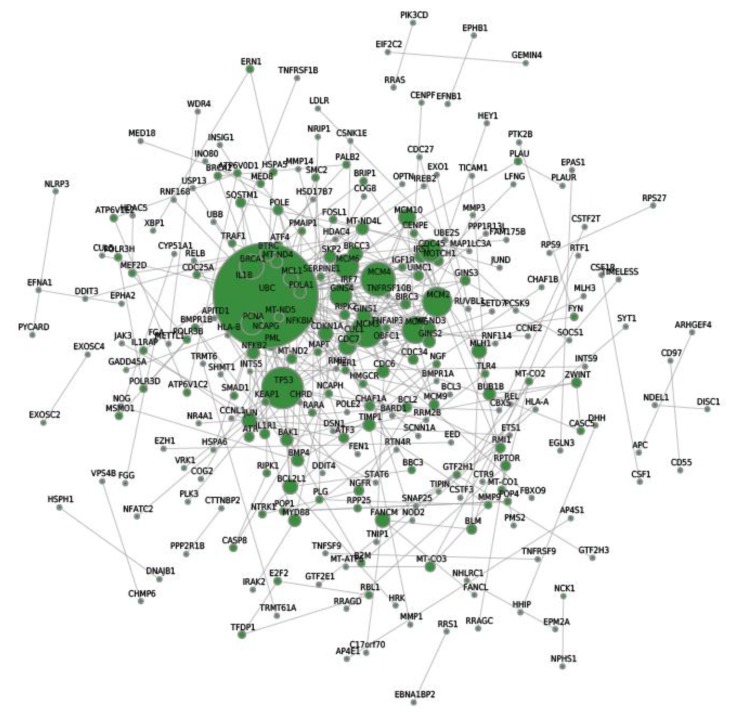
The network of protein–protein interactions (PPI) of differentially expressed genes.

In order to systemically analyze the functions of DEGs in phycocyanin-treated A549 cells, we mapped the DEGs to PPI data and obtained some PPI networks. As shown in Figure 5, a total of 300 relationships between 268 genes (nodes) were identified. Table 4 shows the node genes with the top 20 ranked degree (number of interactions). The DEGs of UBC (ubiquitin-C, degree = 42), TP53 (degree = 16), MCM4 (minichromosome maintenance complex component 4, degree = 13), MCM3 (degree = 12) and MCM2 (degree = 11) formed networks with high degrees. The UBC, TP53, and MCM protein families are important regulation proteins in human cells. They play key roles in multiple cellular activities including cell cycle, DNA replication, apoptotic process, and protein complex assembly [26,27,28,29]. These results indicate that phycocyanin might affect the proliferation of A549 cells through modulating these target genes and classical pathways.

### 2.8. qRT-PCR Validation of Differentially Expressed Genes

**Figure 6:**
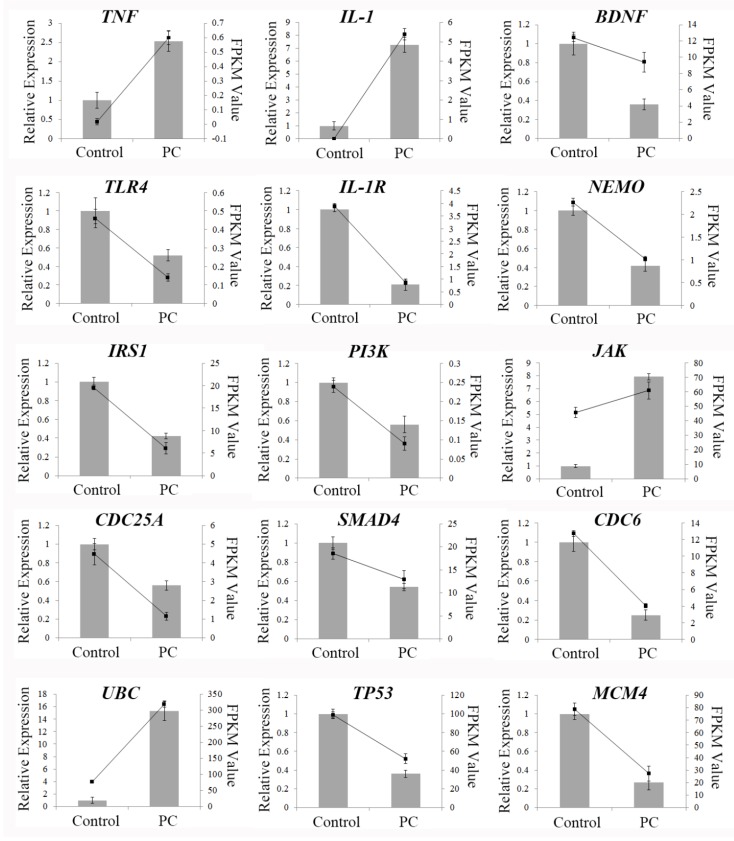
qRT-PCR analysis of the expressions of 15 DEGs. Lines were from the results of the transcriptome data (FPKM Value); column charts were from the results of qRT-PCR (Relative Expression). The expression trends of the detected genes were in accord with those obtained by RNA-seq, including up-regulated genes (TNF, IL-1, JAK, and UBC) and down-regulated genes (BDNF, TLR4, IL-1R, NEMO, IRS1, PI3K, CDC25A, SMAD4, CDC6, TP53, and MCM4). Control: A549 cells without phycocyanin treatment; PC: A549 cells with phycocyanin treatment for 48 h. Three replicates were carried out in the qRT-PCR analysis. Bars represent means ± SD (*n* = 3).

In order to verify the results of transcriptome sequencing and further analyze the expression pattern of genes with important anti-cancer roles, 12 representative genes with different pathways—MAPK signaling (TNF, IL-1, BDNF), NF-κB signaling (TLR4, IL-1R, NEMO), PI3K-AKT signaling (IRS1, PI3K, JAK), cell cycle process (CDC25A, Smad 4, CDC6) from KEGG data, and 3 genes with high protein interaction degrees (UBC, TP53, MCM4) from PPI data—were chosen for quantitative RT-PCR. The qRT-PCR validation results are shown in Figure 6 and their expression trends were found to be in accord with those obtained by RNA-seq, suggesting that the RNA-seq data reliably reflected the gene expression alterations. According to the RNA-seq and qRT-PCR results, the expressions of TNF, IL-1, JAK and UBC increased in phycocyanin-treated A549 cells, while the levels of other genes involved in cell cycle and proliferation processes, including RIPK1, TIRAP, TLR4, TP53, CDC6 and CDC25A, were significantly decreased after phycocyanin treatment. These results were in accord with the cell phenotype experiments.

### 2.9. Pathway Detection in A549 Cells after Phycocyanin Treatment

**Figure 7:**
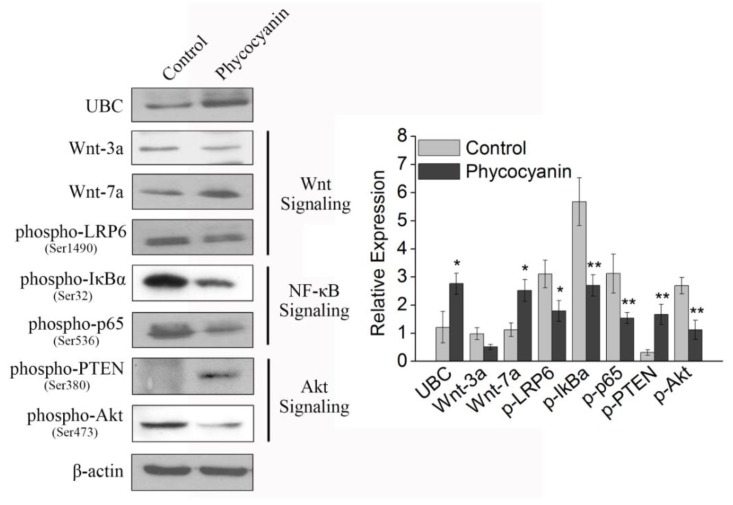
Western blotting analysis of key proteins involved in Wnt, NF-κB and Akt pathways in A549 cells after phycocyanin treatment for 72 h. Control: A549 cells without phycocyanin treatment. The quantification analysis of protein expression is shown in the histogram. Three replicates were carried out in Western blotting analysis (*n* = 3). UBC: ubiquitin-C. Bars represent means ± SD. *, *p* < 0.05; **, *p* < 0.01.

We analyzed some key proteins involved in Wnt (Wnt-3a, Wnt-7a, phospho-LRP6), NF-κB (phospho-IκBα, phospho-p65), and Akt (phospho-PTEN, phospho-Akt) pathways in A549 cells after phycocyanin treatment. As shown in Figure 7, phycocyanin addition increased the expression of UBC in A549 cells, which was in accord with RNA-seq and qRT-PCR results (Figure 6). In addition, the contents of Wnt-3a and phosphorylated LRP6 were decreased, while Wnt-7a, a suppressor of the Wnt pathway, was up-regulated after phycocyanin treatment, indicating that phycocyanin was able to inhibit the activity of Wnt signaling in A549 cells. Moreover, the phosphorylation levels of IκBα, p65, Akt, and PTEN (an inhibitor of Akt signaling) were also significantly altered by phycocyanin (Figure 7). These results suggested that phycocyanin could inhibit multiple pathways in A549 cells, including Wnt, NF-κB, and Akt signaling. Particularly, UBC might play an important regulatory role in this process, which could be a potential mechanism of suppressing cell proliferation by phycocyanin in NSCLC cells.

## 3. Discussion

Several studies have reported the applications of the transcriptome method in cyanobacterium or human cells. Sandrini et al. performed a transcriptome study to investigate cell physiology, gene expression and toxicity of cyanobacterium under high CO_2_ conditions [30], and found that 234 of the 4691 genes responded to elevated CO_2_. Dienst et al. studied the in-depth characterization of the cellular response upon long-term ethanol production in *Synechocystis* sp. PCC6803 using transcriptome technology, and discovered some key genes involved in it [31]. Nevertheless, few studies have reported the antineoplastic mechanism of phycocyanin using the transcriptome method, except Ying et al., who discovered phycocyanin could suppress the proliferation of ovarian cancer cells by regulating VEGF, p53, and neurotrophin pathways [24]. To our knowledge, our work is the first RNA-seq report on the genome expression and potential mechanism of phycocyanin function in NSCLC A549 cells. Subsequently, 2970 differential genes involved in the phycocyanin-mediated inhibition process were analyzed, which participated in different pathways related to anti-tumor functions.

Our work shows that phycocyanin could decrease the viability and proliferation of A549 cells (Figure 1). In fact, several studies have suggested that phycocyanin played dual functions (inhibiting proliferation and inducing apoptosis) in NSCLC cells [16,18]. Besides A549 cells, our previous work also revealed that phycocyanin could suppress proliferation and induce apoptosis in multiple NSCLC cell lines (H1299, H460 and LTEP-a2) [19]. The underlying pro-apoptotic mechanism of action of phycocyanin in NSCLC cells will be investigated in a future study. While the theme of the present work mainly focused on the regulation of cell proliferation ability, the results could help to lay down a theoretical foundation for the application of phycocyanin.

Ubiquitin is a highly conserved, small protein which functions as a tag in the selective proteolysis of abnormal proteins by the 26S proteasome [32]. It could link to target proteins via the activation of the ubiquitin protein ligase. In the present study, ubiquitin-C (UBC) was identified as a differential node protein exerting the highest degree (degree = 42) in the PPI network (Figure 5), which was likely to act as a key regulator in phycocyanin-mediated response in A549 cells.The pathway map retrieved from the KEGG database suggested that NF-κB and TNF signaling were significantly enriched in phycocyanin treated groups (Figure 4). NF-κB comprises a family of transcription factors involved in the regulation of a wide variety of biological responses including cell apoptosis, proliferation and invasiveness [29,30,31]. Phosphorylation of IκBα (an inhibitor of p65) would activate p65 by phosphorylation, resulting in the activation of NF-κB [33,34,35]. The present study indicated that phycocyanin could decrease the activity of NF-κB signaling by down-regulating the phosphorylation levels of p65 and IκBα, which was in accordance with our previous research [19]. Strikingly, UBC could act as an intermediary molecule in TNF-induced NF-κB signaling [36]. Triggering of TNF-R1 by TNF could lead to the recruitment of many cytosolic proteins, which were modified by UBC, and then affected the activity of NF-κB pathway. It is worth noting that the expression trend of TNF and UBC was accordant (both up-regulated) after phycocyanin treatment (Figure 6), which suggested that phycocyanin might modulate the NF-κB pathway through TNF-UBC signaling, resulting in growth inhibition of A549 cells.

Wnt signaling is a central regulatory pathway in controlling key functions of normal and tumor cells, and this has become an important new target for cancer treatment [37]. Wnt/β-catenin is reportedly involved in human malignancies. Upon stimulation with Wnt signaling, LRP6 protein is phosphorylated at multiple sites [37]. Inhibition of Wnt function is associated with decreased cell proliferation and increased apoptosis of NSCLC cells [38]. In our work, Wnt-3 was a down-regulated differential protein (Line 1686 in Supplementary Table S2), suggesting that phycocyanin might inhibit A549 growth via down-regulating Wnt signaling. Moreover, UBC was reported to exert an activation ability of Wnt signaling [39]. Interestingly, Wnt-7a was also identified as a differential protein in our research (Line 392 in Supplementary Table S2), and it was reported that unlike the up-regulation of many Wnt family members, Wnt-7a was down-regulated in most lung cancer cell lines and tumors, acting as a tumor suppressor [40,41]. In this case, the up-regulation of UBC might lead to increased expression of Wnt-7a and decreased phosphorylation level of PLR6, resulting in growth inhibition of A549 cells, which was also probably a potential regulatory mechanism of phycocyanin in NSCLC cells.

As a key factor for stress response, p53 was also significantly enriched as an important node gene in our study. p53 was a cancer suppressor gene closely related to tumor onset and development [42]. It could inhibit the proliferation of tumor cells by triggering cell cycle arrest and apoptosis. It has been reported that p53 has a regulatory effect on NF-κB [43], Wnt [44] and PI3K-AKT [45] signaling, which was significantly enriched in KEGG analysis (Figure 4). In the present study, besides NF-κB and Wnt signaling, phycocyanin could also inhibit PI3K-Akt activity through phosphorylation of PTEN (an inhibitor of Akt signaling) in A549 cells. It is noteworthy that ubiquitin-C had an interaction relationship with p53 translocation in mitochondria [46]. This study suggested that UBC/p53 was involved in A549 cell growth inhibition triggered by phycocyanin. Transcriptome has provided an efficient way to discover the potential mechanism of phycocyanin. Ying et al. performed RNA-seq in ovarian cancer cell model, and found that the MAPK/p53 pathway plays a key role in phycocyanin-mediated growth inhibition of SKOV-3 cells [24]. Unlike the work by Ying et al., we focused on the upstream of regulatory processes. Our results revealed that UBC was an important upstream regulator in A549 cells after phycocyanin treatment [19]. It could interact with p53 and participate in regulating multiple pathways including Wnt, NF-κB and Akt signaling. The present study helps to reveal the regulatory mechanism of phycocyanin in NSCLC cells comprehensively.

**Figure 8:**
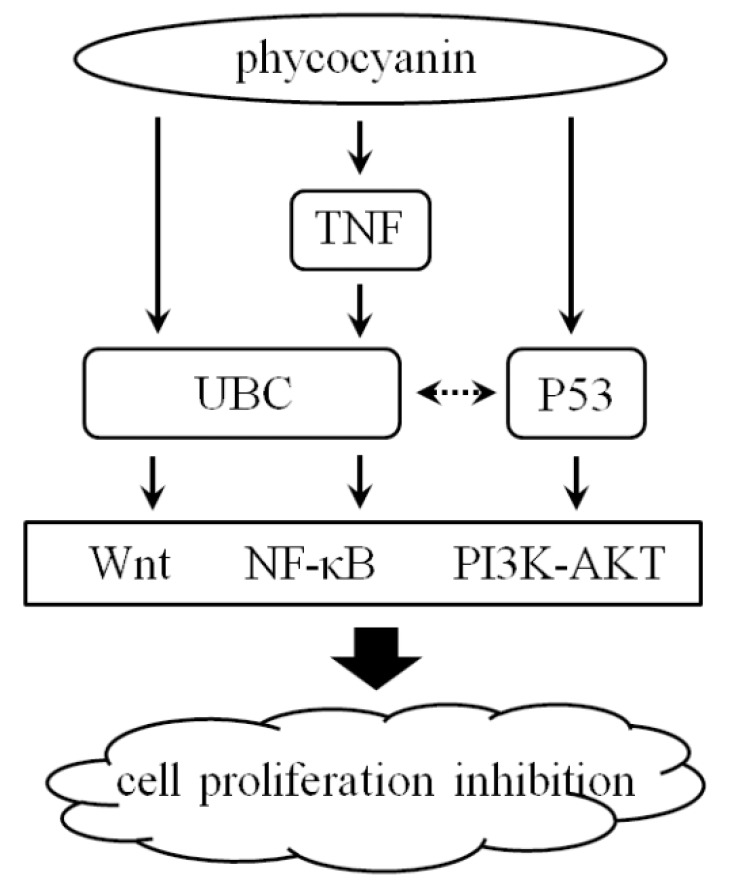
Illustration of potential regulatory mechanism of phycocyanin in NSCLC A549 cells.

In conclusion, phycocyanin displayed a significant suppression effect on the proliferation of NSCLC A549 cells, which was a result of interactions among multiple pathways and signal molecules. Interfering a series of signal pathways including TNF, NF-κB and Wnt, and some key node factors such as UBC and p53, phycocyanin influenced the growth of A549 cells (Figure 8). This work systematically analyzed the potential mechanism of phycocyanin in NSCLC A549 cells, and laid the foundation for further application research of phycocyanin.

## 4. Materials and Methods

### 4.1. Materials, Cell Line and Culture Conditions

Phycocyanin (derived from *Spirulina platensis*) standard substance was purchased from Envirologix (Portland, ME, USA). Phycocyanin was dissolved in phosphate buffer solution (PBS) according to the specification. Human NSCLC cell line A549 was purchased from American Type Cell Collection (ATCC, Manassas, VA, USA). Cells were cultured in DMEM media supplemented with 10% heat-inactivated fetal bovine serum (FBS), 0.1 mg/mL of streptomycin, and 100 units/mL of penicillin at 37 °C in a humidified atmosphere with 5% CO_2_. Cells were sub-cultured every 3–4 days. Cells between 3–12 passages were used in the experiments.

### 4.2. Cell Survival Rate Assay

The cell survival rate was analyzed by MTT method as described in our former study [19]. Briefly, A549 cells were seeded at a density of 5000 cells in 100 µL of complete medium per well into 96-well plates. After overnight incubation, phycocyanin (0, 1.2, 2.4, 4.8 and 9.6 µM) was added to each well. In our study, the control cells (0 µM) are A549 cells treated with equivalent PBS as phycocyanin treatment cells. Each condition was tested using four replicates. After incubation for 24 h, the culture medium was supplemented with 1 mg/mL MTT for 4 h at 37 °C. The medium was then removed, and the cells were solubilized with DMSO. The absorbance was measured at 450 nm and 630 nm. The results were expressed as a percentage of the absorbance reading of the control cells.

### 4.3. Cell Proliferation Assay

Cell proliferation was determined by MTT method as described in our previous work [19]. Briefly, A549 cells were seeded at 5000 cells in 100 µL of complete medium per well in quadruplicate in 96-well plates. After 12 h of incubation for cell attachment, phycocyanin was added into each well with a final concentration of 0, 1.2, 2.4, and 4.8 µM, respectively. Each day, 10 µL MTT/well was added to test cells and incubated for 4 h at 37 °C. Then SDS-HCl solution (10% SDS, 0.01M HCl) was added into each well and incubated for 14 h at 37 °C. The absorbance of formazan was measured at a wavelength of 570 nm. The assay lasted for 5–6 days after treatment. Three independent experiments were performed.

### 4.4. RNA Extraction and cDNA Library Construction

Total RNA was extracted using Trizol reagent at 4 °C, and the quantity was determined by 1% agarose gel electrophoresis. The RNA samples with qualified purity (OD_260_/OD_280_ ≥ 1.8, OD_260_/OD_230_ ≥ 1.5) were obtained using microscale-ultraviolet spectrophotometer. The quality and purity of the RNA samples were further assessed using an RNA 6000 Nano LabChip Kit and a Bioanalyzer 2100 (Agilent Technologies, Santa Clara, CA, USA), using an RNA integrity number (RIN) of ≥ 8.0. Poly-(A)-containing mRNA was purified using oligo (dT) magnetic beads and an Oligotex mRNA Kit (Qiagen, Hilden, Germany). Fragmentation buffer was added to disrupt the mRNA strands into short fragments, which were used as templates to synthesize the first-strand cDNA using reverse transcriptase and random hexamer primers. The second-strand cDNA was synthesized using buffer, dNTPs, RNase H, and DNA polymerase I. The double-strand cDNA fragments were subjected to end repair and adapter ligation. Adapter-modified fragments were selected using gel purification and PCR amplified to create the final cDNA library.

### 4.5. Transcriptome Sequencing and Bioinformatics Analysis

Transcriptome sequencing was performed using the high-throughput sequencing platform of Illumina HiSeq 4000 (Illumina, San Diego, CA, USA). 5 µg of total RNA was used for RNA-seq analysis. Base calling was adopted to convert original sequencing images to sequential data. The raw reads were subjected to adapter trimming and low quality filtering using Trimmomatic program [47]. The high quality clean reads were aligned to the human genome using TopHat [48]. Human genome sequence and gene annotation were obtained from the UCSC Genome Website. Cuffdiff was used to profile differentially expressed genes with default parameters [49]. All unigenes were queried against commonly used databases using BLASTx search to identify homologs (E-value < 10^−10^). The databases used were Swiss-prot [50], KEGG [51], Nr [52], KOG [53], and GO [54]. The differentially expressed genes (DEGs) between control and phycocyanin-treated A549 cell line were identified based on fragments per kilobases per millionreads (FPKM) using RSEM 1.2.31 [55]. DESeq was used to determine the FDR (false discovery rate) threshold (adjust *p* value). If the FDR was less than 0.05 in the multi-group comparison, it was considered to be a significantly different expression level.

### 4.6. Quantitative RT-PCR (qRT-PCR) on Gene Expressions

Total RNAs from A549 cells without phycocyanin treatment and from those treated with 4.8 µM phycocyanin for 48 h were used for qRT-PCR analysis. 2 µg of total RNA was reverse transcribed and quantified with a SYBR Green real-time PCR Master Mix Kit (Takara, Dalian, China). GAPDH was used as an endogenous control. The primers were shown in Supplementary Table S2. The relative expression of each gene was calculated and normalized using the 2^−ΔΔCt^ method relative to GAPDH. Each assay was performed in quadruplicate.

### 4.7. Western Blotting

The cells were lysed using RIPA lysate (1% NP40, 0.1% SDS, 5 mM EDTA, 0.5% sodium deoxycholate, and 1 mM sodium orthovanadate). Equal amounts (20 µg) of the samples were separated by SDS-PAGE and then transferred to a polyvinylidene fluoride membrane. The following primary antibodies (Cell Signaling Technology, Boston, MA, USA) were incubated with the membrane at 4 °C overnight: anti-ubiquitin-C (1:1000 dilution); anti-Wnt-3a (1:1000); anti-Wnt-7a (1:2000); anti-phospho-LRP6 (1:1000); anti-phospho-IκBα (1:1000); anti-phospho-p65 (1:1000); anti-phospho-PTEN (1:500); anti-phospho-Akt (1:1000) and anti-β-action (1:2000) antibodies. Secondary antibodies (Cell Signaling Technology, Boston, MA, USA, 1:5000 dilution) were added and incubated with enhanced chemiluminescence (ECL) reagent (including Luminol and Peroxide solution, Millipore, Schwalbach, Germany). The chemiluminescence, which reflected the expression of proteins, was visualized by X-ray film.

### 4.8. Statistical Analysis

Numerical data were expressed as means ± standard deviation (SD). The difference between means was analyzed using SPSS 16.0 software (SPSS Inc., Chicago, IL, USA). Two-tailed Student’s *t*-tests were performed for comparison among groups. In addition, *p* < 0.05 (*) or *p* < 0.01 (**) was considered as statistically significant.

## Figures and Tables

**Table 1:** Summary of trimming and read mapping results of the sequences generated from A549 cells with or without phycocyanin treatment.

Sample	Raw Reads	Clean Reads	Total Mapped	Multiple Mapped	Uniquely Mapped
Contorl-1	71,748,960	66,148,142	64,387,922 (97.34%)	2,099,216 (3.17%)	62,288,706 (94.17%)
Control-2	70,062,692	64,302,644	62,557,790 (97.29%)	2,054,749 (3.2%)	60,503,041 (94.09%)
Control-3	54,566,932	52,411,510	51,378,836 (98.03%)	1,624,560 (3.1%)	49,754,276 (94.93%)
PC-1	58,518,666	56,346,378	55,114,355 (97.81%)	2,257,228 (4.01%)	52,857,127 (93.81%)
PC-2	51,947,736	49,626,788	48,530,748 (97.79%)	1,928,329 (3.89%)	46,602,419 (93.91%)
PC-3	61,563,182	59,220,992	58,041,957 (98.01%)	2,122,980 (3.58%)	55,918,977 (94.42%)

Note: Control represents A549 cells without phycocyanin treatment; PC represents A549 cells with phycocyanin treatment for 48 h. Three replicates of control (control-1, -2 and -3) and PC (PC-1, -2 and -3) treatments were carried out in RNA-seq analysis.

**Table 2:** Functional annotation of transcriptome data in three public protein databases.

Type	Transcript Number (percent)	Gene Number (percent)
COG	16,917 (8.49%)	16,890 (29%)
GO	70,127 (35.2%)	19,197 (9.64%)
KEGG	104,630 (52.52%)	13,266 (6.66%)
Total annotation	122,847 (61.66%)	19,937 (10.01%)
Total	199,234	58,233

**Table 3:** Number of differentially expressed genes (DEGs) at adjusted *p* ≤ 0.05, and |log2fc| fold change ≥ 1: Within and between genotype comparisons for A549 cells with and without phycocyanin treatment.

	Total	Up	Down
Total DEGs	18,639	9670	8969
Significant DEGs	2970	1539	1431

**Table 4:** Node genes with top 20 ranked degree in the PPI network.

Tanscript/Gene ID	String ID	Degree
ENSG00000150991	UBC	42
ENSG00000141510	TP53	16
ENSG00000104738	MCM4	13
ENSG00000112118	MCM3	12
ENSG00000073111	MCM2	11
ENSG00000100297	MCM5	10
ENSG00000076003	MCM6	9
ENSG00000012048	BRCA1	9
ENSG00000147536	GINS4	8
ENSG00000132646	PCNA	8
ENSG00000093009	CDC45	8
ENSG00000097046	CDC7	6
ENSG00000065328	MCM10	6
ENSG00000101003	GINS1	5
ENSG00000076242	MLH1	5
ENSG00000124762	CDKN1A	5
ENSG00000171552	BCL2L1	5
ENSG00000187790	FANCM	5
ENSG00000055130	CUL1	5
ENSG00000143384	MCL1	5

Note: Degree represents the numbers of protein–protein interactions.

## Supplementary Materials

The following are available online at http://www.mdpi.com/1660-3397/16/12/511/s1. Supplementary Table S1. Detailed information of differentially expressed genes (DEGs); Supplementary Table S2. Primers used for Quantitative RT-PCR; Figure S1. Clusters Orthologous Groups of proteins (COG) annotation of A549 cell genes and transcripts; Figure S2. Histogram presentation of gene distribution in Gene Ontology (GO) functional classification; Figure S3. KEGG analysis of genes in A549 cells after treatment with phycocyanin for 48 h.

## References

[1] Jemal A., Bray F., Center M.M., Ferlay J., Ward E., Forman D. (2011). Global cancer statistics. CA Cancer J. Clin..

[2] Liu F., Wang C., Hu T., Wang W. (2018). S-1-based concurrent chemoradiotherapy in the treatment of locally advanced non-small cell lung cancer: A systematic review and meta-analysis protocol. Medicine.

[3] Carrizosa D.R., Gold K.A. (2015). New strategies in immunotherapy for non-small cell lung cancer. Transl. Lung Cancer Res..

[4] Chen Y.Y., Huang T.W., Tsai W.C., Lin L.F., Cheng J.B., Chang H., Lee S.C. (2014). Risk factors of postoperative recurrences in patients with clinical stage I NSCLC. World J Surg. Oncol..

[5] Liu Q., Huang Y., Zhang R., Cai T., Cai Y. (2016). Medical application of Spirulina platensis derived C-phycocyanin. Evid. Based Complement. Alternat. Med..

[6] Li B., Chu X., Gao M., Li W. (2010). Apoptotic mechanism of MCF-7 breast cells in vivo and in vitro induced by photodynamic therapy with C-phycocyanin. Acta Biochim. Biophys. Sin..

[7] Thangam R., Suresh V., Asenath Princy W., Rajkumar M., Senthilkumar N., Gunasekaran P., Rengasamy R., Anbazhagan C., Kaveri K., Kannan S. (2013). C-Phycocyanin from Oscillatoria tenuis exhibited an antioxidant and in vitro antiproliferative activity through induction of apoptosis and G0/G1 cell cycle arrest. Food Chem..

[8] Nemoto-Kawamura C., Hirahashi T., Nagai T., Yamada H., Katoh T., Hayashi O. (2004). Phycocyanin enhances secretary IgA antibody response and suppresses allergic IgE antibody response in mice immunized with antigen-entrapped biodegradable microparticles. J. Nutr. Sci. Vitaminol..

[9] Jensen G.S., Attridge V.L., Beaman J.L., Guthrie J., Ehmann A., Benson K.F. (2015). Antioxidant and anti-inflammatory properties of an aqueous cyanophyta extract derived from Arthrospira platensis: Contribution to bioactivities by the non-phycocyanin aqueous fraction. J. Med. Food.

[10] Eriksen N.T. (2008). Production of phycocyanin—A pigment with applications in biology, biotechnology, foods and medicine. Appl. Microbiol. Biotechnol..

[11] Liao G.Y., Gao B., Gao Y.N., Yang X.G., Cheng X.D., Ou Y. (2016). Phycocyanin inhibits tumorigenic potential of pancreatic cancer cells: Role of apoptosis and autophagy. Sci. Rep..

[12] Saini M.K., Sanyal S.N. (2014). Targeting angiogenic pathway for chemoprevention of experimental colon cancer using C-Phycocyanin as cyclooxygenase-2 inhibitor. Biochem. Cell Biol..

[13] Liu Z.J., Fu X., Huang W., Li C.X., Wang X.Y., Huang B. (2018). Photodynamic effect and mechanism study of selenium-enriched phycocyanin from Spirulina platensis against liver tumours. J. Photochem. Photobiol..

[14] Minic S.L., Stanic-Vucinic D., Mihailovic J., Krstic M., Nikolic M.R., Cirkovic Velickovic T. (2016). Digestion by pepsin releases biologically active chromopeptides from C-phycocyanin, a blue-colored biliprotein of microalga Spirulina. J. Proteomics.

[15] Li B., Gao M.H., Chu X.M., Teng L., Lv C.Y., Yang P., Yin Q.F. (2015). The synergistic antitumor effects of all-trans retinoic acid and C-Phycocyanin on the lung cancer A549 cells in vitro and in vivo. Eur. J. Pharmacol..

[16] Li B., Gao M.H., Lv C.Y., Yang P., Yin Q.F. (2016). Study of the synergistic effects of all-transretinoic acid and C-Phycocyanin on the growth and apoptosis of A549 cells. Eur. J. Cancer Prev..

[17] Baudelet P.H., Gagez A.L., Berard J.B., Juin C., Bridiau N., Kaas R., Thiery V., Cadoret J.P., Picot L. (2013). Antiproliferative activity of Cyanophora paradoxa pigments in melanoma, breast and lung cancer cells. Mar. Drugs.

[18] Bingula R., Dupuis C., Pichon C., Berthon J.Y., Filaire M., Pigeon L., Falaire E. (2016). Study of the effects of betaine and/or C-Phycocyanin on the growth of lung cancer A549 cells in vitro and in vivo. J. Oncol..

[19] Hao S., Yan Y., Li S., Zhao L., Zhang C., Liu L.Y., Wang C.T. (2018). The in vitro anti-tumor activity of phycocyanin against non-small cell lung cancer cells. Mar. Drugs.

[20] Tang F., Barbacioru C., Wang Y., Nordman E., Lee C., Xu N., Wang X., Bodeau J., Tuch B.B., Siddiqui A. (2009). mRNA-seq whole-transcriptome analysis of a single cell. Nat. Methods.

[21] Wilhelm B.T., Marguerat S., Goodhead I., Bähler J. (2010). Defining transcribed regions using RNA-seq. Nat. Protoc..

[22] Jian J., Wei W., Yin G., Hettinghouse A., Liu C., Shi Y. (2018). RNA-seq analysis of interferon inducible p204-mediated network in anti-tumor immunity. Sci. Rep..

[23] Aldaz C.M., Hu Y., Daniel R., Gaddis S., Kittrell F., Medina D. (2002). Serial analysis of gene expression in normal p53 null mammary epithelium. Oncogene.

[24] Ying J., Wang J., Ji H., Lin C., Pan R., Zhou L., Song Y., Zhang E., Ren P., Chen J. (2016). Transcriptome analysis of phycocyanin inhibitory effects on SKOV-3 cell proliferation. Gene.

[25] Subhashini J., Mahipal S.V., Reddy M.C., Mallikarjuna Reddy M., Rachamallu A., Reddanna P. (2004). Molecular mechanisms in C-Phycocyanin induced apoptosis in human chronic myeloid leukemia cell line-K562. Biochem. Pharmacol..

[26] Wiborg O., Pedersen M.S., Wind A., Berglund L.E., Marcker K.A., Vuust J. (1985). The human ubiquitin multigene family: Some genes contain multiple directly repeated ubiquitin coding sequences. EMBO J..

[27] Macheret M., Halazonetis T.D. (2015). DNA replication stress as a hallmark of cancer. Annu. Rev. Pathol..

[28] Suzuki S., Kurata M., Abe S., Miyazawa R., Murayama T., Hidaka M., Yamamoto K., Kitagawa M. (2012). Overexpression of MCM2 in myelodysplastic syndromes: Association with bone marrow cell apoptosis and peripheral cytopenia. Exp. Mol. Pathol..

[29] Samel S.A., Fernandez-Cid A., Sun J., Riera A., Tognetti S., Herrera M.C., Li H., Speck C. (2014). A unique DNA entry gate serves for regulated loading of the eukaryotic replicative helicase MCM2-7 onto DNA. Genes Dev..

[30] Sandrini G., Cunsolo S., Schuurmans J.M., Matthijs H.C., Huisman J. (2015). Changes in gene expression, cell physiology and toxicity of the harmful cyanobacterium Microcystis aeruginosa at elevated CO_2_. Front. Microbiol..

[31] Dienst D., Georg J., Abts T., Jakorew L., Kuchmina E., Borner T., Wilde A., Duhring U., Enke H., Hess W.R. (2014). Transcriptomic response to prolonged ethanol production in the cyanobacterium Synechocystis sp. PCC6803. Biotechnol. Biofuels..

[32] Ciechanover A., Heller H., Elias S., Haas A.L., Hershko A. (1980). ATP-dependent conjugation of reticulocyte proteins with the polypeptide required for protein degradation. Proc. Natl. Acad. Scie. USA.

[33] Dolcet X., Llobet D., Pallares J., Matias-Guiu X. (2005). NF-kB in development and progression of human cancer. Virchows Arch..

[34] Deveraux Q.L., Roy N., Stennicke H.R., Van Arsdale T., Zhou Q., Srinivasula S.M., Alnemri E.S., Salvesen G.S., Reed J.C. (1998). IAPs block apoptotic events induced by caspase-8 and cytochrome c by direct inhibition of distinct caspases. EMBO J..

[35] Guttridge D.C., Albanese C., Reuther J.Y., Pestell R.G., Baldwin A.S. (1999). NF-kappaB controls cell growth and differentiation through transcriptional regulation of cyclin D1. Mol. Cell. Biol..

[36] Verhelst K., Verstrepen L., Carpentier I., Beyaert R. (2011). Linear ubiquitination in NF-kappaB signaling and inflammation: What we do understand and what we do not. Biochem. Pharmacol..

[37] Tai D., Wells K., Arcaroli J., Vanderbilt C., Aisner D.L., Messersmith W.A., Lieu C.H. (2015). Targeting the WNT Signaling Pathway in Cancer Therapeutics. Oncologist.

[38] Stewart D.J. (2014). Wnt signaling pathway in non-small cell lung cancer. J. Natl. Cancer Inst..

[39] Han W., Lee H., Han J.K. (2017). Ubiquitin C-terminal hydrolase37 regulates Tcf7 DNA binding for the activation of Wnt signalling. Sci. Rep..

[40] Tennis M.A., Vanscoyk M.M., Wilson L.A., Kelley N., Winn R.A. (2012). Methylation of Wnt7a is modulated by DNMT1 and cigarette smoke condensate in non-small cell lung cancer. PLoS ONE.

[41] Winn R.A., Marek L., Han S.Y., Rodriguez K., Rodriguez N., Hammond M., Van Scoyk M., Acosta H., Mirus J., Barry N. (2005). Restoration of Wnt-7a expression reverses non-small cell lung cancer cellular transformation through frizzled-9-mediated growth inhibition and promotion of cell differentiation. J. Biol. Chem..

[42] Bates S., Vousden K.H. (1999). Mechanisms of p53-mediated apoptosis. Cell. Mol. Life Sci..

[43] Lowe J.M., Menendez D., Bushel P.R., Shatz M., Kirk E.L., Troester M.A., Garantziotis S., Fessler M.B., Resnick M.A. (2014). p53 and NF-kappaB coregulate proinflammatory gene responses in human macrophages. Cancer Res..

[44] Kim N.H., Kim H.S., Kim N.G., Lee I., Choi H.S., Li X.Y., Kang S.E., Cha S.Y., Ryu J.K., Na J.M. (2011). p53 and microRNA-34 are suppressors of canonical Wnt signaling. Sci. Signal..

[45] Abraham A.G., O’Neill E. (2014). PI3K/Akt-mediated regulation of p53 in cancer. Biochem. Soc. Trans..

[46] Marchenko N.D., Wolff S., Erster S., Becker K., Moll U.M. (2007). Monoubiquitylation promotes mitochondrial p53 translocation. EMBO J..

[47] Bolger A.M., Lohse M., Usadel B. (2014). Trimmomatic: A flexible trimmer for Illumina sequence data. Bioinformatics.

[48] Trapnell C., Pachter L., Salzberg S.L. (2009). TopHat: Discovering splice junctions with RNA-seq. Bioinformatics.

[49] Trapnell C., Hendrickson D.G., Sauvageau M., Goff L., Rinn J.L., Pachter L. (2013). Differential analysis of gene regulation at transcript resolution with RNA-seq. Nat. Biotechnol..

[50] Mortazavi A., Williams B.A., McCue K., Schaeffer L., Wold B. (2008). Mapping and quantifying mammalian transcriptomes by RNA-seq. Nat. Methods.

[51] Livak K.J., Schmittgen T.D. (2001). Analysis of relative gene expression data using real-time quantitative PCR and the 2(-Delta Delta C(T)) Method. Methods.

[52] Srikanth A., Schmid M. (2011). Regulation of flowering time: All roads lead to Rome. Cell. Mol. Life Sci..

[53] Boeckmann B., Bairoch A., Apweiler R., Blatter M.C., Estreicher A., Gasteiger E., Martin M.J., Michoud K., O’Donovan C., Phan I. (2003). The SWISS-PROT protein knowledgebase and its supplement TrEMBL in 2003. Nucleic Acids Res..

[54] Ogata H., Goto S., Sato K., Fujibuchi W., Bono H., Kanehisa M. (1999). KEGG: Kyoto encyclopedia of genes and genomes. Nucleic Acids Res..

[55] Krylov D.M., Wolf Y.I., Rogozin I.B., Koonin E.V. (2003). Gene loss, protein sequence divergence, gene dispensability, expression level, and interactivity are correlated in eukaryotic evolution. Genome Res..

