# Supplementary material for: Transcriptome Analysis of Phycocyanin-Mediated Inhibitory Functions on Non-Small Cell Lung Cancer A549 Cell Growth
Source: Mar Drugs. 2018 Dec 15;16(12):511. doi: 10.3390/md16120511 (PMC6316159; doi:10.3390/md16120511)
Supplement: Supplementary file 1 [file marinedrugs-16-00511-s001.zip › suppl/Supplementary Material_Figures.pdf]

Transcriptome Analysis of Phycocyanin-Mediated Inhibitory Functions on Non-Small Cell Lung Cancer A549 Cell Growth

Shuai Hao[1], Shuang Li[1], Jing Wang[1], Lei Zhao[1], Yan Yan[1], Qi Cao[1], Tingting Wu[1], Liyun Liu[2,*] and Chengtao Wang[1,*]

[1] Beijing Advanced Innovation Center for Food Nutrition and Human Health, Beijing Engineering and Technology Research Center of Food Additives, Beijing Technology and Business University, Beijing, 100048, China

[2] State Key Laboratory of Infection Disease Prevention and Control, National Institute for Communicable Disease Control and Prevention, Collaborative Innovation Center for Diagnosis and Treatment of Infectious Disease, Chinese Center for Disease Control and Prevention, Beijing, 102206, China

* Correspondence: ctwangbtbu@163.com; liuliyun@icdc.cn

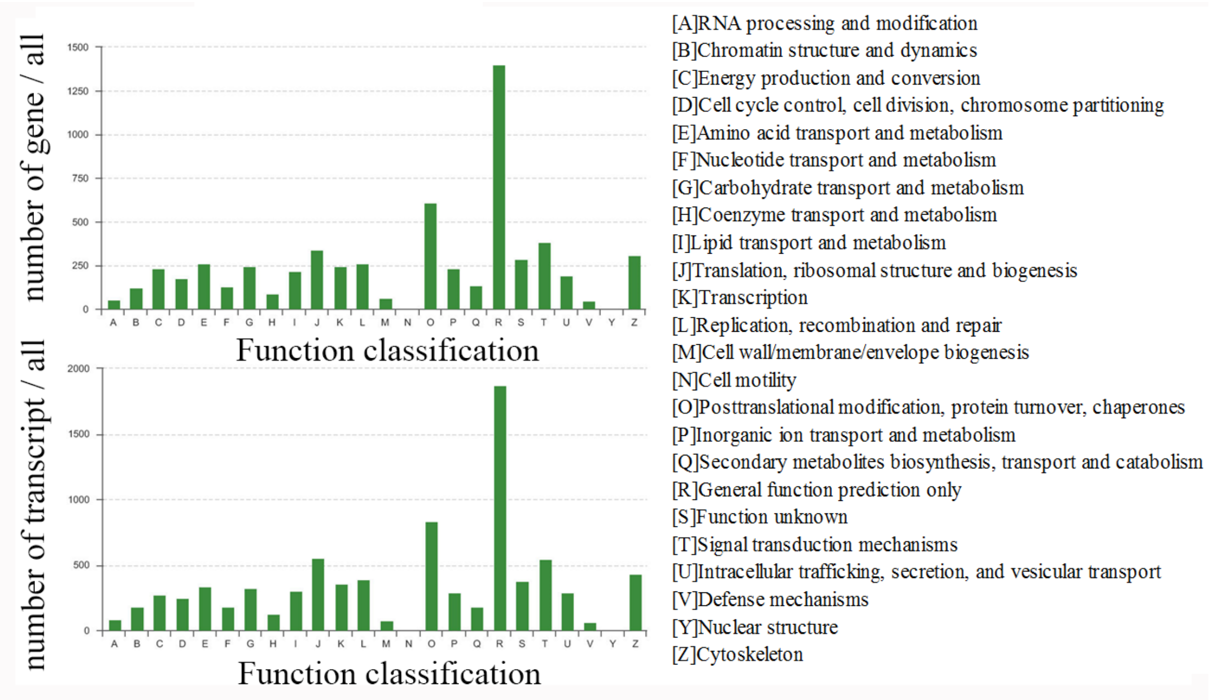

Figure S1. Clusters Orthologous Groups of proteins (COG) annotation of A549 cell genes and transcripts.

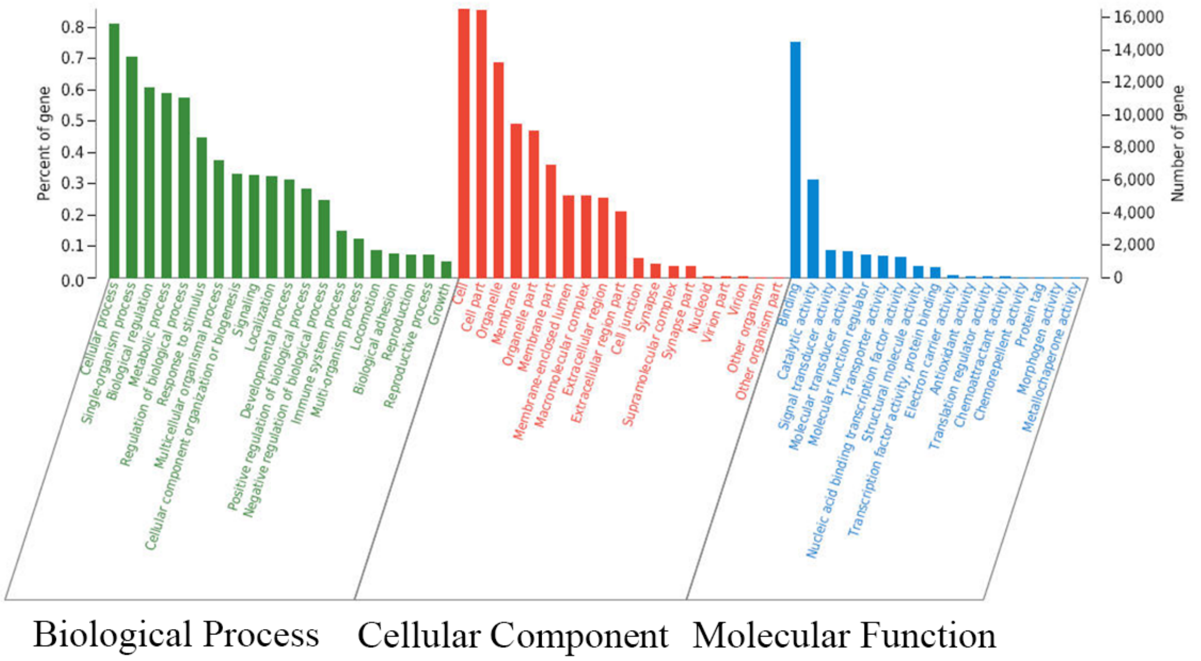

Figure S2. Histogram presentation of gene distribution in Gene Ontology (GO) functional classification. Genes were further classified into sub-groups in biological process, cellular component and molecular function.

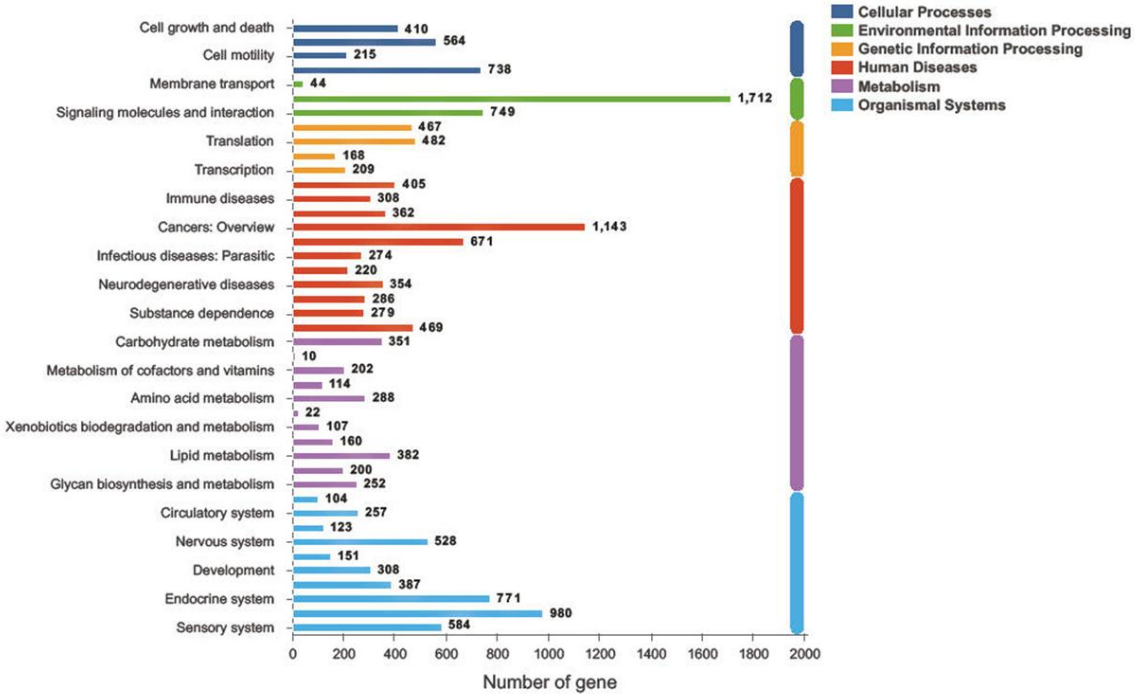

Figure S3. KEGG analysis of genes in A549 cells after treatment with phycocyanin for 48 h.
